# Sex-Specific ADNP/NAP (Davunetide) Regulation of Cocaine-Induced Plasticity

**DOI:** 10.1007/s12031-024-02234-2

**Published:** 2024-09-10

**Authors:** Yael Toren, Yarden Ziv, Shlomo Sragovich, R. Anne McKinney, Segev Barak, Shula Shazman, Illana Gozes

**Affiliations:** 1 The Elton Laboratory for Molecular Neuroendocrinology, Department of Human Molecular Genetics and Biochemistry, Faculty of Medicine, Sagol School of Neuroscience and Adams Super Center for Brain Studies, Tel Aviv University, Tel Aviv, 6997801 Israel; 2 School of Psychological Sciences, Sagol School of Neuroscience, Tel Aviv University, Tel Aviv, 6997801 Israel; 3 Department of Pharmacology and Therapeutics, McGill University, Montreal, Canada; 4 Department of Mathematics and Computer Science, The Open University of Israel, Ra’anana, Israel

**Keywords:** Cocaine, Addiction, ADNP, NAP, Structural plasticity, Sex differences

## Abstract

Cocaine use disorder (CUD) is a chronic neuropsychiatric disorder estimated to effect 1–3% of the population. Activity-dependent neuroprotective protein (ADNP) is essential for brain development and functioning, shown to be protective in fetal alcohol syndrome and to regulate alcohol consumption in adult mice. The goal of this study was to characterize the role of ADNP, and its active peptide NAP (NAPVSIPQ), which is also known as davunetide (investigational drug) in mediating cocaine-induced neuroadaptations. Real time PCR was used to test levels of *Adnp* and *Adnp2* in the nucleus accumbens (NAc), ventral tegmental area (VTA), and dorsal hippocampus (DH) of cocaine-treated mice (15 mg/kg). Adnp heterozygous *(Adnp* ^+/−^)and wild-type *(Adnp* ^+/−^) mice were further tagged with excitatory neuronal membrane-expressing green fluorescent protein (GFP) that allowed for *in vivo* synaptic quantification. The mice were treated with cocaine (5 injections; 15 mg/kg once every other day) with or without NAP daily injections (0.4 µg/0.1 ml) and sacrificed following the last treatment. We analyzed hippocampal CA1 pyramidal cells from 3D confocal images using the Imaris x64.8.1.2 (Oxford Instruments) software to measure changes in dendritic spine density and morphology. *In silico* ADNP/NAP/cocaine structural modeling was performed as before. Cocaine decreased *Adnp* and *Adnp2* expression 2 h after injection in the NAc and VTA of male mice, with mRNA levels returning to baseline levels after 24 h. Cocaine further reduced hippocampal spine density, particularly synaptically weaker immature thin and stubby spines, in male *Adnp*^+/+^) mice while increasing synaptically stronger mature (mushroom) spines in *Adnp*^+/−^) male mice and thin and stubby spines in females. Lastly, we showed that cocaine interacts with ADNP on a zinc finger domain identical to ketamine and adjacent to a NAP-zinc finger interaction site. Our results implicate ADNP in cocaine abuse, further placing the ADNP gene as a key regulator in neuropsychiatric disorders. Ketamine/cocaine and NAP treatment may be interchangeable to some degree, implicating an interaction with adjacent zinc finger motifs on ADNP and suggestive of a potential sex-dependent, non-addictive NAP treatment for CUD.

## Introduction

Cocaine use disorder (CUD) is a chronic disorder characterized by compulsive drug-seeking and consumption, as well as symptoms of craving and withdrawal (Schwartz et al. 2022). Cocaine exposure can cause rapid and long-lasting changes in the central nervous system, disrupting learning and memory processes and promoting addictive behavior (Nestler 2001). These cocaine-induced neuroadaptations include changes in gene expression (Fernandez-Castillo et al. 2022), synaptic plasticity (Spronk et al. 2013), and structural remodeling at the level of the dendritic spine, the main site of excitatory synapses (Dos Santos et al. 2017; Dumitriu et al. 2012). For example, cocaine increases spine density in medium spiny neurons of the nucleus accumbens (NAc) (Robinson and Kolb 1999), and these changes can be rapid (Madangopal 2017), sub-regional, and sub-type specific (Dumitriu et al. 2012) and induced by even a single dose of cocaine (Shen et al. 2009).

From a neurochemical point of view, a 2019 study showed that microtubule (MT) end binding protein 3 (EB3), essential for dendritic spine formation, mediates structural and behavioral adaptation during withdrawal from cocaine self-administration (Calipari et al. 2019). The same study also suggested the involvement of the actin cytoskeletal system. A separate work by Damuka et al. demonstrated changes in MTs *in vitro*, *in vivo*, and *ex vivo* in a rodent model of CUD, further linking the cytoskeletal system with cocaine (Damuka et al. 2022).

In both human and animal models, there is evidence for biologically based sex differences underlying CUD (Fattore et al. 2020; Becker 2016). Using quantitative proteomics, a recent study found that cocaine induces non-overlapping protein expression patterns in male and females, with differences in drug-associated proteins GRM2, VPS51, SATT, VGLU3, IPO4, VAMP1, and CYFP2 (Lopez et al. 2021). A systematic review further puts the solute carrier family 6, neurotransmitter transporter, serotonin, member 4 (*SLC6A4*) as a major gene common to addiction, depression, and anxiety (Kaushik et al. 2023).

First discovered in Professor Illana Gozes laboratory, activity-dependent neuroprotective protein (ADNP) is an essential protein for brain development and cognitive functioning (Bassan et al. 1999; Zamostiano et al. 2001). ADNP is a leading autism spectrum disorder (ASD)-linked gene (Helsmoortel et al. 2014) and is further associated with Alzheimer’s disease (Gozes 2024), schizophrenia (Dresner et al. 2011), and stress response (Sragovich et al. 2019a, b) and is protective in fetal alcohol syndrome (Poggi et al. 2003; Pascual and Guerri 2007). Recently, we presented ADNP as an alcohol-responsive gene and negative regulator of alcohol consumption in adult female mice (Ziv et al. 2019), and the question arises whether ADNP is also involved in cocaine use disorder.

Significantly, ADNP regulates many cellular processes that could be relevant to CUD, including transcription, and autophagy (Sragovich et al. 2017). As part of the SWI/SNF chromatin remodeling complex, ADNP regulates transcription of over 400 genes (Mandel et al. 2008), including *SLC6A4* (Amram et al. 2016), and P13-K/Akt, which activate several intracellular signaling pathways (Pascual and Guerri 2007; Hacohen-Kleiman et al. 2018; Karmon et al. 2022) involved in drug addiction (Cao et al. 2016). Its protective activity on nerve cells is largely mediated through its NAP motif (single amino acid code, NAPVSIPQ, also called CP201 or davunetide) interacting with MT end-binding proteins EB1 and EB3 (Oz et al. 2014).

In clinical studies, NAP, the ADNP active peptide fragment, has been shown to protect cognitive and functional activity in patients with mild cognitive impairment (Morimoto et al. 2013), as well as in schizophrenia patients (Vaisburd et al. 2015), in women suffering from progressive supranuclear palsy (PSP) (Gozes et al. 2023), and is currently under development for the treatment of ADNP syndrome (Gozes 2024). These data, along with favorable brain bioavailability and safety profile (Sragovich et al. 2019a, b; Gozes et al. 2005), position NAP as a promising drug candidate for neuropsychiatric disorders.

Notably, we (the Gozes laboratory) discovered extensive sex-dependent deficiencies in ADNP-mutated mice (Gozes 2017), coupled with sex-dependent effects on dendritic spines, axonal transport, and tubulin isotype expression (Amram et al. 2016; Hacohen-Kleiman et al. 2018; Karmon et al. 2022; Sragovich et al. 2019a, b; Vulih-Shultzman et al. 2007). These include the findings that hippocampal ADNP transcript are doubled in male vs. female *Adnp*^+/−^ mice, with male *Adnp*^+/−^ mice exhibiting greater impairments in object recognition and social memory (Malishkevich et al. 2015). Additionally, NAP treatment increased the expression of *Nlgn2* and *Nlgn3* (neuroligins) in the young *Adnp*^*+/*−^ male mouse cortex, while it decreased these transcripts in the older *Adnp*^*+/−*^ male with no effect in females, suggesting that ADNP/NAP are involved in sexually dichotomous brain plasticity (Hacohen-Kleiman et al. 2018). Importantly, we discovered significant increase in males (but not females) of the hippocampal *Slc6a4* levels in the *Adnp*^*+/−*^, compared with *Adnp*^*+/+*^ mice. Treatment with the NAP EB1/EB3 active site SKIP reduced *Slc6a4* expression levels in the hippocampus of the *Adnp*^*+/−*^ mice to control levels, whereas the control peptide, D-SKIP, did not (Amram et al. 2016).

Taken together with ADNP promotion of sex-dependent neuronal morphogenesis/plasticity (Bennison et al. 2023), ADNP-mediated regulation of steroid biosynthesis genes (Grigg et al. 2020), sexually dichotomized (Malishkevich et al. 2015), estrous cycle (Furman et al. 2005), and gonadotropin-releasing hormone receptor (*Gnrhr*)-correlated ADNP expression (Kapitansky et al. 2020) all imply potential sex-dependent association of ADNP with cocaine addiction.

In 2001, the Gozes laboratory first described ADNP2, as a family member paralogue/homologue to ADNP (Zamostiano et al. 2001), further revealing ADNP2 protection against oxidative stress (Kushnir et al. 2008). ADNP/ADNP2 control of erythropoiesis (Dresner et al. 2012) and ADNP/ADNP2 coregulation (Giladi et al. 2007; Malishkevich et al. 2015) with dysregulation association with aging (Kapitansky and Gozes 2019), as well as brain disorders, such as schizophrenia (Dresner et al. 2011; Merenlender-Wagner et al. 2015) and Alzheimer’s disease (Malishkevich et al. 2016). Additionally, a de novo single nucleotide variation in ADNP2 has been associated with developmental abnormalities (Chung et al. 2015), and CpG hypermethylation of ADNP2 has been linked with post-traumatic stress disorder (PTSD) (Bainomugisa et al. 2021).

In this study, we tested whether cocaine exposure affects *Adnp*/*Adnp2* expression in the mesolimbic system in wild-type mice. We next utilized the *Adnp*-deficient mouse model (Hacohen-Kleiman et al. 2018) to investigate the role of ADNP/NAP in cocaine-induced plasticity, namely, dendritic spine content and structure, in a sex dependent manner. We then demonstrated that cocaine (like ketamine; Ganaiem et al. 2023) and NAP show *in silico* affinity to adjacent ADNP Zn finger domains. Finally, we review possible mechanisms through which ADNP might mediate cocaine-response and conclude by discussing implications of the role of ADNP/NAP in cocaine addiction for future prevention and treatment.

## Methods and Materials

### Animals

All protocols conformed the guidelines of the Institutional Animal Care and Use Committee of Tel Aviv University and the Israeli Ministry of Health, as well as the guidelines of the NIH (animal welfare assurance number A5010-01). All efforts were made to minimize the number of animals used. All animals were housed in Tel-Aviv University Animal Facility (12-h light-dark cycle—lights on at 4 a.m., food, and water ad libitium). C57BL/6J mice (25–30 g) were housed 3–4/cage; *Adnp*^*+/+*^ and littermates, *Adnp*^*+/−*^ mice (outbred with ICR strain for 30 generations in Tel-Aviv University Animal Facility; 25–30 g) (Sragovich et al. 2019a, b; Hacohen-Kleiman et al. 2018; Ziv et al. 2019) were individually housed. Bedding was standard sterilized dust-saw, and rodent chow used were Teklad Global 18% protein (Envigo, Israel).

### Cocaine Administration by Intraperitoneal (IP) Injection

Three-month-old male and female mice of ICR background were bred with a second strain of C57BL mice which are transgenic for the GFP gene (Chang et al. 2014), resulting in *Adnp*^*+/+*^***/***^*+/−*^*GFP* black mice.

The mice first underwent 2 days of handling, followed by 3 days of habituation in which they were injected with saline. Following habituation, the mice were treated with either cocaine 15 mg/kg (10 ml/kg) or saline (10 ml/kg) and a secondary treatment included either 4 µg NAP (0.1 ml/per animal) or 0.1 ml saline for 5 days, every other day. This is consistent with an intermittent injection schedule which is known to produce sensitization, a process thought to underlie drug craving and relapse (Oliveira-Lima et al. 2017; Steketee 2005). This protocol is consistent with previously published work (Gaval-Cruz et al. 2012; Goltseker et al. 2017). All of the mice were sacrificed immediately after the fifth session, and their brains were processed for further analysis.

### Tissue Collection

Brains were quickly removed from euthanized mice and placed on an ice-cold platform. Tissues from the nucleus accumbens (NAc), dorsal hippocampus (DH), and ventral tegmental area (VTA), brain regions having a major role in the mesolimbic system, which is strongly linked to reward and addiction, were carefully dissected from 1-mm slices using a brain matrix and immediately snap-frozen in liquid nitrogen and stored at − 80 °C until use. Further analysis included quantitative reverse transcription-real-time polymerase chain reaction (qRT-PCR), as previously described (Ziv et al. 2019) and illustrated below.

### RNA Extraction from Mouse NAc, VTA, and DH Samples (Ziv et al. 2019)

Different brain areas were kept inside 1.5 ml RNAase free vials.  A 500 µl of TRIzol reagent were added to the samples along with mechanical crushing using a plastic pestle to breakdown the brain cells. A 100 µl of chloroform were added to separate the RNA from DNA and cell debris. The samples underwent centrifugation, and 150–200 µl of the top clear layer were taken from each vial. Each sample was mixed with 900 µl of ethanol 100% and 30 µl of sodium acetate and was incubated for 4 h in -20 °C. After 4 h, the samples underwent another centrifugation and were washed using 750 µl of ethanol 70%. Each sample was dried and re-suspended in 20 µl of ultra-pure water. A 280 µl of ultra-pure water were added to all samples, and the samples were mixed again with 900 µl of ethanol 100% and 30 µl of sodium acetate and were incubated in -20 °C overnight. The day after the above procedures, the samples were washed and dried again as described earlier (Krebs et al. 2009).

### Real-time PCR (Ziv et al. 2019)

The expression levels of the *Adnp*,* Adnp2*, and glyceraldehyde-3-phosphate dehydrogenase (*Gapdh)* mRNA species were analyzed by quantitative real time PCR.

Reverse transcription was performed, using the total RNA that was extracted from the mouse brain samples. The amplification was done by using SYBR Green PCR mix. The relative quantity of the specific mRNA expression was determined, using the ΔΔCt method, and the changes in expression were normalized to *Gapdh*.

The primers that were used for amplification are the following:

- *Adnp*: F (ACGAAAAATCAGGACTATCGG)
  R (GGACATTCCGGAAATGACTTT)
- *Adnp2*: F (GGAAAGAAAGCGAGATACCG)
  R (TCCTGGTCAGCCTCATCTTC)
- *Gapdh*: F (CCAGAACATCATCCCTGC)
  R (GGAAGGCCATGCCAGTGAGC)

*Gapdh* is a common reference gene for measuring mRNA expression (Dundas and Ling 2012), frequently used in cocaine studies (Fischer et al. 2022; Caffino et al. 2011; Fumagalli et al. 2006). As explained in previous work (Ziv et al. 2019), there is no reference gene appropriate for all experiments, and future studies can further explore this.

### Dendritic Spine Quantification and Analysis

Three-month-old mice of ICR background were bred with a second strain of C57BL mice, which are transgenic for the GFP gene, to produce an *Adnp*^*+/–*^- mGFP mouse model (Chang et al. 2014; Paola et al. 2003). The breeding resulted in *Adnp*^*+/+*^***/***^*+/-*^*GFP* black mice. Mice were divided to groups according to sex, genotype, primary treatment (cocaine/vehicle), and secondary treatment (NAP/saline). Experiment duration was 9 days: On days 1, 3, 5, 7, and 9, the mice received cocaine (15 mg/kg, 10 ml/kg), or saline I.P. injection (10 ml/kg) and a second injection of either 4 µg NAP (0.1 ml/per animal) or 0.1 ml saline. On the days when the mice did not participate in a session, an injection of either 4 µg NAP (0.1 ml/per animal) or 0.1 ml saline was administered in the home cages. On day 9, mice were perfused, and brains were extracted (Hacohen-Kleiman et al. 2018). We analyzed CA1 pyramdial cells ~ 6–40 tertiary dendrites per animal from 3D confocal images, using the Imaris x64.8.1.2 (Oxford Instruments) software. Data were analyzed by normalizing the number of spines to dendrite length and group pooling of all animal results.

### Statistical Analysis

Results are described as means ± standard error of the mean (SEM). Data generated from qRT-PCR experiments was analyzed by one-way ANOVA, with a group factor (2–24 h after cocaine injection). LSD post-hoc analysis followed significant effects. Gene expression levels were normalized to *Gapdh* expression, and each brain region was normalized to its own control. For the dendritic spine data, two-way ANOVA with Tukey’s post hoc was conducted to see effect of genotype x treatment on total spine density and subtypes (ANOVA; SPSS 14). To compare effects of treatment within the same genotype, one-way ANOVA with Tukey’s post hoc was performed. Additional analyses that compared only two groups, for example, male and female data for each group, were performed using unpaired Student’s t-test (SPSS 14). *P* values of < 0.05 were deemed statistically significant (*p < 0.05, **p < 0.01, ***p < 0.001).

### *In silico *modeling 

I-TASSER (https://zhanggroup.org/I-TASSER) was used for protein structure modelling, and HDOCK (http://hdock.phys.hust.edu.cn/) was used for *in silico* protein/protein docking of ADNP, cocaine (structure taken from pdb : 1I7Z), ketamine (as before (Ganaiem et al. 2023), and NAP. PatchFinderPlus (PFplus) (https://bindup.technion.ac.il/) (Shazman et al. 2007) was used for electrostatic calculations. PyMOL software was used to create figures.

## Results

### Cocaine Administration by Intraperitoneal (IP) Injection Causes an Apparent Short-Term Decrease in *Adnp* and *Adnp2* mRNA Levels in Male Mice

**Fig. 1:**
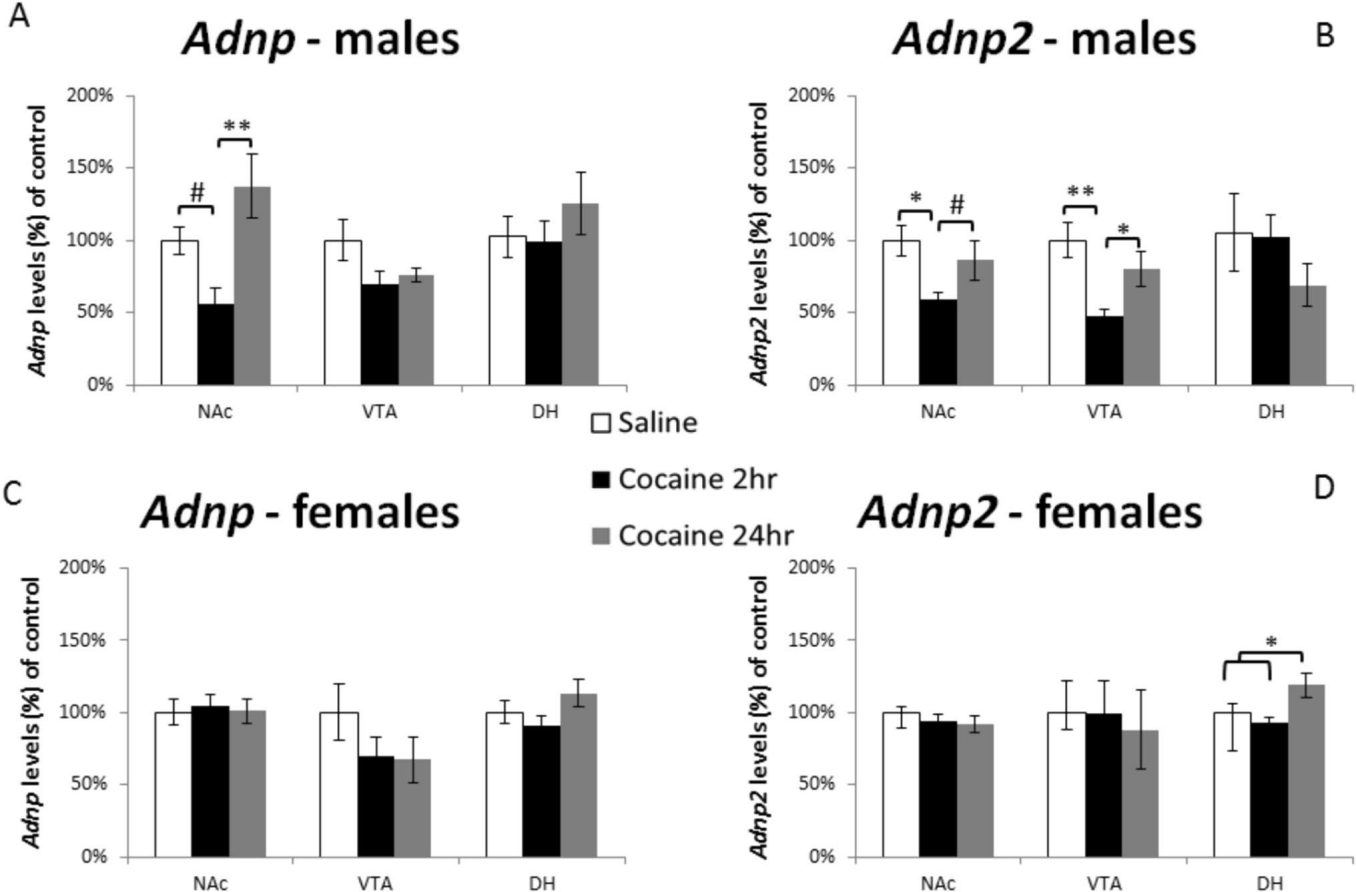
Cocaine administration affects ***Adnp*** and ***Adnp2*** relative expression following cocaine injections in males and female mice; 3-month-old male and female mice were administered with either 15 mg/kg of cocaine or saline (10 ml/kg)﻿ 3 times every other day. At the age of 4 months, the mice were given an additional injection and were sacrificed either 2 h after the injection or 24 h later. The brains were removed, and the NAc, VTA, and DH were recovered. Each brain region’s results are normalized to its own saline control. *Adnp* and *Adnp2* expression was determined using real time PCR. Results are expressed as mean ± SEM and normalized to *Gapdh* #*p* < 0.1, *p < 0.05, **p < 0.01, ***p < 0.001. (*n* = 5–6/per group). **A** One-way ANOVA shows a trend of decrease in *Adnp* expression in the male NAc 2 h post-injection (F(2,15) = 6.897, *p* = 0.008) (LSD post hoc, *p* = 0.062) followed by an increase back to basal levels, 24 h later. No effects were found in the VTA or DH (*p > 0.05). **B ***Adnp2* levels were significantly reduced in both male NAc and VTA, 2 h after the cocaine injection (NAc: F(2,15) = 4.120, *p* = 0.037, LSD post hoc, *p* = 0.013; VTA: F(2,13) = 7.681, *p* = 0.006, LSD post hoc, *p* = 0.002). No significant difference was detected in the DH (*p > 0.05). **C** In female mice, there was no change in *Adnp* expression in the tested regions (*p > 0.05), **D** but *Adnp2* was increased in the DH 24 h after injection (F(2,15) = 4.646, *p* = 0.03, LSD post hoc, *p* = 0.046)

We first tested how cocaine affects *Adnp* and *Adnp2* expression in wild-type C57BL mice. Cocaine primarily targets the mesolimbic system; therefore, we focused on three mesolimbic brain regions: NAc, VTA, and DH. As shown in Fig. 1, in male mice, we saw a reduction of *Adnp* mRNA levels in the NAc (Fig. 1A**)** and of *Adnp2* in the NAc and VTA (Fig. 1B**)** 2 h after the last cocaine injection, with a return to baseline after 24 h. In contrast, in female mice, we saw no change in *Adnp* expression in the NAc, VTA, or DH at either time point (Fig. 1C**)**, but there was a significant increase of *Adnp2* expression in the DH 24 h after cocaine treatment (Fig. 1D).

#### Cocaine and NAP Control Dendritic Spine Growth in a Spine- and Sex-Dependent Manner

**Fig. 2:**
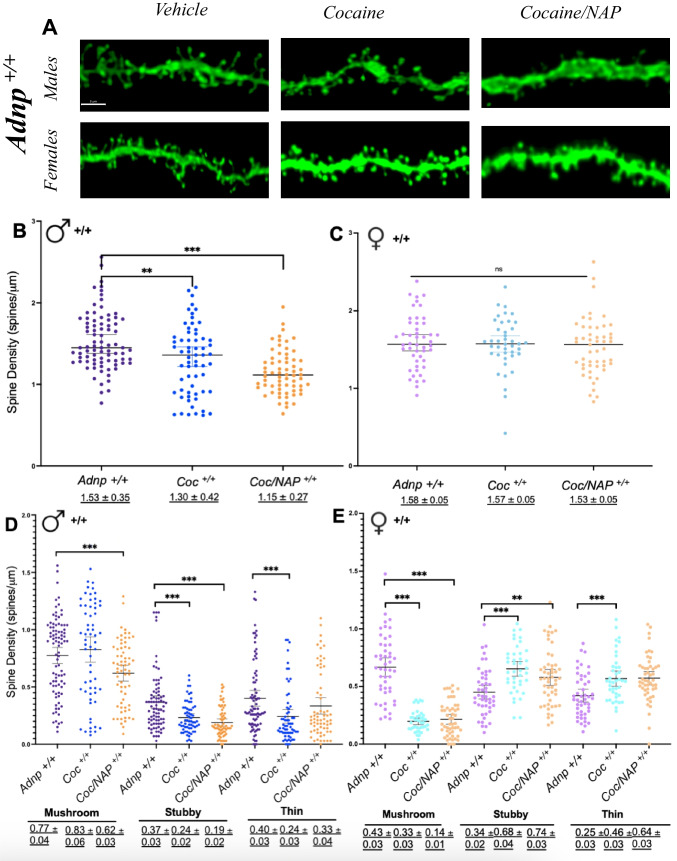
Sub-chronic cocaine and cocaine/NAP exposure decreases total hippocampal spine density in male Adnp^***+/+***^ mice, with no change in female mice. In both sexes, cocaine treatment is associated with morphological changes. Each of the experimental groups described below included 3–4 independent mice and the total number of dendritic spines counted per the entire experimental group is delineated below (analyzing ~ 6–40 tertiary dendrites/mouse). **A**  Representative image of GFP-labeled CA1 pyramidal neuron dendritic spines from Adnp^*+/+*^ male or female mice treated with control vehicle, cocaine, or cocaine + NAP. Scale bar = 3 μm. Average total spine density in Adnp^*+/+*^ mice (males,  Adnp^*+/+*^ *n*  = 75, Adnp^*+/+*^  cocaine *n*  = 66, Adnp^*+/+*^ NAP + cocaine *n*  = 69; females,  Adnp^*+/+*^ *n*  = 48, Adnp^*+/+*^  cocaine *n*  = 41, Adnp^*+/+*^  cocaine + NAP *n*  = 51). A two-way ANOVA with Tukey’s post hoc test was performed, followed by one-way ANOVA to determine treatment effects within Adnp^*+/-*^ groups. Additional Student’s t test was performed to determine sex differences. Underlined numbers beneath the graphs represent the mean ± SEM. **B**  For male Adnp^*+/+*^ mice, there was a main effect of treatment on total spine density [F(3,271) = 16.051, ***p < 0.001], **D**  as well as on mushroom [F(3,271) = 4.470, **p < 0.005], stubby [F(3,271) = 10.482, ***p < 0.001], and thin [F(3,272) = 6.812, ***p < 0.001] subtypes. Tukey’s post-hoc revealed a significant effect on total spine density of cocaine (***p  < 0.001) and combined cocaine/NAP (**p < 0.001) treatment, as well as significant differences in stubby and thin subtype with cocaine (***p = 0.001) and cocaine/NAP (***p  < 0.001). **C** For female Adnp^*+/+*^ mice, there was no effect of treatment on total spine density. [F(3,185) = 0.1940, p  = 0.9]. **E** However, there was a significant effect of treatment on mushroom [F(3,184) = 73.913, p < 0.001], stubby [F(3,184) = 6.748, ***p < 0.001], and thin [F(3,183) = 14.569, ***p  < 0.001] spine subtypes

Given that both cocaine (Calipari et al. 2019) and ADNP/NAP are known to regulate spine dynamics (Hacohen-Kleiman et al. 2018; Karmon et al. 2022), we then measured the effects of cocaine and cocaine/NAP on dendritic spines in *Adnp*-deficient (*Adnp*^*+/−*^) versus wild-type mice (*Adnp*^*+/+*^) (Fig. 2A). We found that in *Adnp*^*+/+*^ mice, both cocaine and cocaine/NAP treatment, reduced total spine density in males (Fig. 2B, ***p < 0.001), with no change in female mice (Fig. 2C, *p* = 0.9). The NAP direct effects were studied before and published, showing no treatment effect in females and a spine number reducing effect in males, coupled with significantly increased protective shaft synapse volumes (Hacohen-Kleiman et al. 2018).

Changes in spine morphology have been suggested to represent differences in their function (Yuste and Bonhoeffer 2001), with thin spines seen as transient/immature (“learning”) spines and mushroom spines considered stable/mature (“memory”) spines (Hering and Sheng 2001; Bourne and Harris 2007). For this reason, further measurements were made for dendritic spine subgroups classified on the basis of the following morphology types: stubby spines (< 0.5 *µm* in length, lacking a clear head), mushroom spines (mushroom-shaped head, approximately 1 *µm* in length), thin spines (with an elongated narrow neck with a distinctive head), and filopodia (McKinney 2010). Our results showed that cocaine selectively reduced stubby (Fig. 2D, **p < 0.01) and thin (***p < 0.001) spines in male mice, with cocaine/NAP treatment decreasing mushroom-type spines. In female *Adnp*^*+/+*^ mice, cocaine also reduced mushroom spines (Fig. 2E, ***p < 0.001) but increased stubby and thin spines (***p < 0.001), thereby keeping the total spine density stable.

#### Cocaine Increases Dendritic Spine Density in *Adnp*-Deficient Mice

**Fig. 3:**
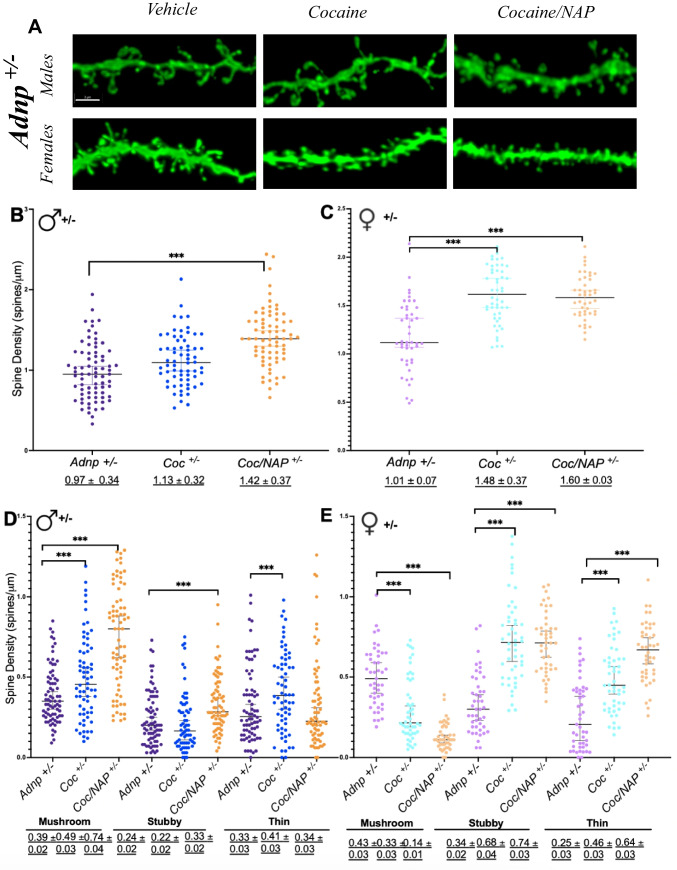
Adnp^***+/−***^ mice have decreased hippocampal spine density, ameliorated by both cocaine and NAP treatment. Notably, in male Adnp^*+/−*^ mice, there is a synergistic effect of combined treatment, with cocaine/NAP resulting in the greatest increase. Each of the experimental groups described below included 4–5 independent mice, and the total number of dendritic spines counted per the entire experimental group is delineated below (analyzing ~ 6–40 tertiary dendrites/mouse). **A** Representative image of GFP-labeled CA1 pyramidal neuron dendritic spines from Adnp^*+/−*^ male or female mice treated with control vehicle, cocaine or cocaine + NAP. Scale bar = 3 μm. Average total spine density in Adnp^*+/–*^ males (Adnp^*+/–*^ [*n* = 75, Adnp^*+/–*^ cocaine *n* = 66, Adnp^*+/–*^ NAP + cocaine *n* = 69) and females (Adnp^*+/+*^ *n* = 45, Adnp^*+/−*^ *n*  = 23, Adnp^*+/−*^ cocaine *n* = 50, Adnp^*+/−*^ cocaine + NAP n  = 44). A two-way ANOVA with Tukey’s post hoc test was performed, followed by one-way ANOVA to determine treatment effects within Adnp^*+/−*^ groups. Additional Student’s t test was performed to determine sex differences. Underlined numbers beneath graphs represent the mean ± SEM. **B** In male mice, for total spine density, main genotype [F(1,545) = 23.479, **p < 0.001] and interaction [F(3,545) = 6.388, ***p < 0.001] effects were found. **C** In female mice, for total spine density, main genotype [F(1,231) = 59.957, ***p < 0.001] and treatment [F(3,231) = 27.516, * **p <  0.001] effects were found. Tukey’s post hoc revealed significant differences between cocaine (***p < 0.001) versus vehicle-treated mice. **D** For male Adnp *+/–* mice, 1-way ANOVA revealed a main effect of treatment on mushroom [F(3,278) = 1.649, ***p < 0.001] and stubby [F(3,277) = 5.737, *** *p* = 0.001] spines. Tukey’s post hoc revealed significant differences in mushroom and stubby spines density between cocaine/NAP-treatment versus cocaine alone (mushroom ***p < 0.001, stubby *p* = 0.003) treatment alone. **E** For females, a main effect for treatment was seen for mushroom [F(3,184) = 77.173, ***p  < 0.001], stubby [F(3,184) = 52.076, ***p  < 0.001], and thin [F(3,184) = 1.258, ***p < 0.001] spines

*Adnp*^*+/−*^ mice have reduced dendritic spine density as compared to wild-type mice (Vulih-Shultzman et al. 2007), which is ameliorated by NAP treatment (see Hacohen-Kleiman et al. 2018 for full NAP results). Here, we found that (like NAP) cocaine treatment increases hippocampal spine density in male and female *Adnp*^*+/−*^ mice (Fig. 3A, B, C). Interestingly, in male mice, there was an additive (synergistic) effect of cocaine and NAP, with combined treatment resulting in the greatest increase in spine density (***p < 0.001) and complete normalization.

Mushroom spines in particular are reduced with *Adnp* deficiency and increased by NAP treatment (Hacohen-Kleiman et al. 2018). Building on these data, we showed that cocaine similarly increases mushroom spines in male *Adnp*^*+/−*^ mice (Fig. 3D, ***p < 0.001), with combined cocaine/NAP treatment showing the greatest increase (***p < 0.001). The opposite was true in female *Adnp*^*+/−*^ mice, with cocaine decreasing mushroom spines (Fig. 3E, ***p < 0.001) and combined cocaine/NAP further reducing mushroom density but increasing stubby/thin spine subtypes (***p < 0.001). That cocaine/NAP initiate similar forms of structural remodeling (i.e., bias toward stable, mushroom spines in male mice, and toward immature thin/stubby spines in females) could reflect the induction of shared, sex-dependent signaling pathways.

#### Cocaine and NAP Show *In Silico* Affinity to Adjacent ADNP Zinc Finger Domains

**Fig. 4:**
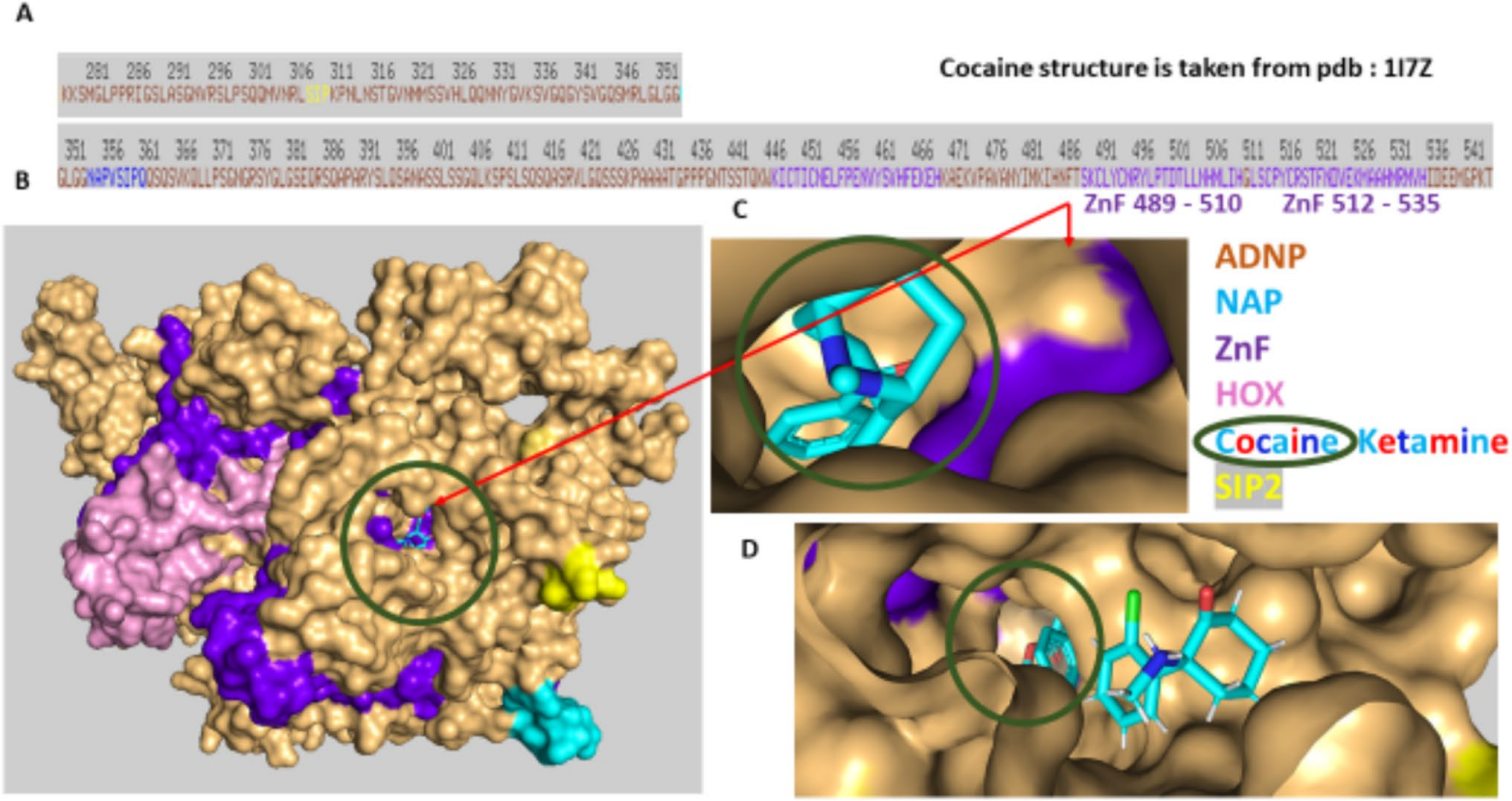
Cocaine interacts with ADNP on a zinc finger domain identical to ketamine. Experimental means are delineated in the methods section. **A** The ruler on top indicates the human ADNP linear amino acid sequence at the site of ketamine binding (red single letter amino acid code). The NAP motif (NAPVSIPQ, amino acids, 354–361, on ADNP) is indicated in blue (or cyan), including the EB1/EB3 binding SIP sequence. The second ADNP-SIP motif ADNP amino acids, 308–310 (SIP2), is also shown on the ruler in yellow. Zinc fingers (Znf) are shown in purple. **B** ADNP, surface view, and cocaine circled in green sticks are shown (HOX, pink, represents the ADNP homeobox domain) (Zamostiano et al. 2001; Ganaiem et al. 2023). **C** Represents an enlargement of (**A**) focusing on the Znf. **D** Zooming in on cocaine (circled in green) and ketamine (sticks) interactions with ADNP

Lastly, we used *in silico* modeling to explore possible cocaine/NAP interactions. Our most recent results (Ganaiem et al. 2023) partly explain the NAP effects on gene expression, showing NAP nuclear penetrance and a NAP direct interaction with an ADNP zinc finger domain (Ganaiem et al. 2023). Given the fact that cocaine may partly regulate ADNP expression (Fig. 1) and that ADNP auto-regulates its own synthesis (Mandel et al. 2007; Aboonq et al. 2012), we investigated *in silico* interactions of cocaine and ADNP. Our results identified cocaine proximity and presumptive interaction with a zinc finger. The identified zinc finger was localized on ADNP amino acids 489–510 (Fig. 4A, ADNP sequence, Fig. 4B an overall view of cocaine-ADNP interaction, Fig. 4C, zooming in on ADNP-cocaine interaction, ZnF denotes zinc finger).

**Fig. 5:**
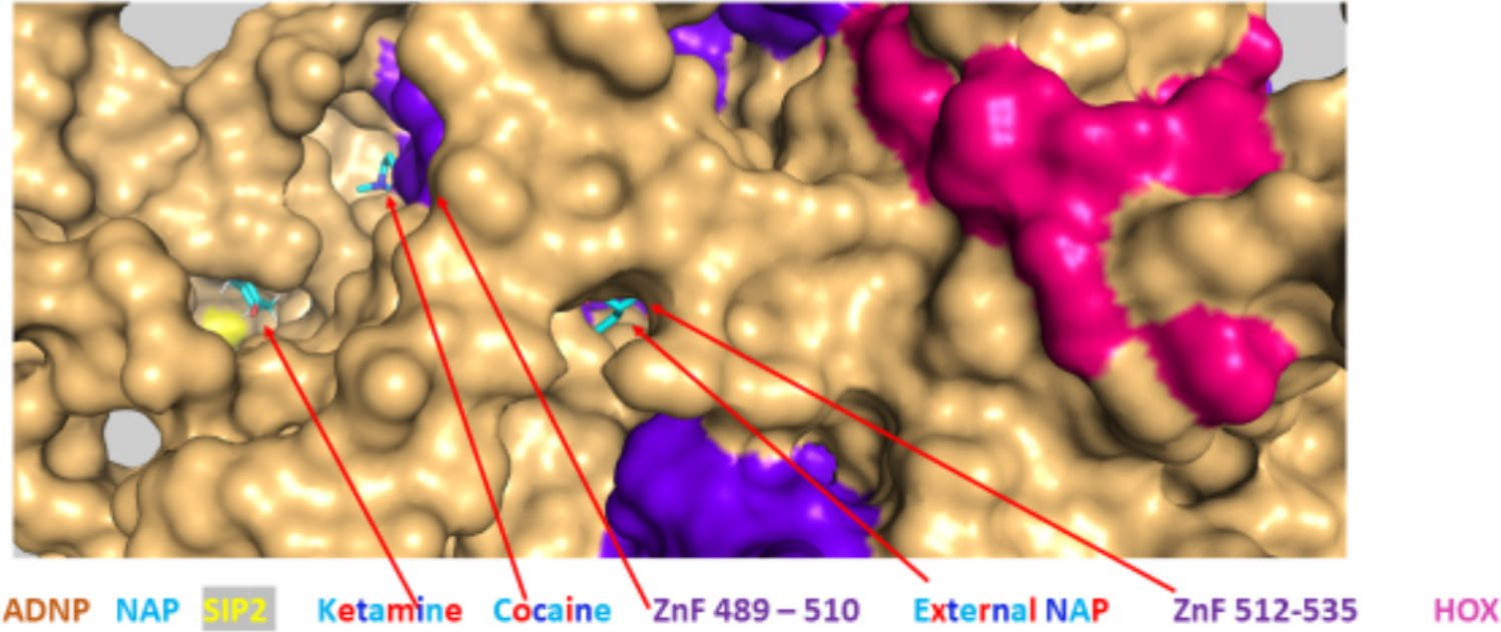
Cocaine interacts with ADNP on a zinc finger domain identical to ketamine and adjacent to a NAP-zinc finger interaction site. ADNP surface view enlarged with cocaine, ketamine, and NAP docking (details are delineated in the methods and in Fig. 4 legend)

Interestingly, this specific ZnF also interacts with ketamine (Ganaiem et al. 2023) (Fig. 4D), which in turn interacted also with the second EB1/EB3 interacting SIP motif (except for NAP), denoted SIP2 (yellow highlights) (see also Fig. 5). Figure 5 tested for NAP interactions in the presence of cocaine (and compared to ketamine) showing proximity amongst these different molecules on the ADNP surface, with NAP closest proximity to the adjacent ZnF, namely amino acids, 512–535 on ADNP.

## Discussion

Here, we introduce ADNP and its active peptide fragment, NAP, as a novel regulator of cocaine-induced plasticity. Our finding that cocaine differentially changes *Adnp*/*Adnp2* transcript levels in male vs. female mice adds to a growing literature describing sex differences in cocaine use (Becker 2016) and highlight genetic and synaptic changes underlying these differences.

Previously, we showed that *Adnp* and *Adnp2* levels are increased in the dorsal hippocampus of male and female mice and in the NAc of females, following repeated alcohol injection (Ziv et al., 2019). In contrast, cocaine caused a short-term decrease in *Adnp* and *Adnp2* mRNA levels in male, but not female mice. Taken together, current data support the role of ADNP in mediating substance abuse in a drug- and sex-dependent manner.

The fact that we found similar dendritic phenotypes after cocaine and NAP (Hacohen-Kleiman et al. 2018) suggests that both activate shared (sex-dependent) pathways to modulate spine growth (as evidenced by increased thin spines) and maturation (as evidenced by increased mushroom spines) in CA1 pyramidal cells in the hippocampus. Spine stabilization is dependent on the entry of MT-EB3 plus ends into dendritic spine heads, suggesting that cocaine/NAP may differentially effect MT dynamics in male versus female mice. This agrees with our results on robust sex differences in a novel Adnp genome-edited mouse model, carrying the most abundant ADNP heterozygous pathologic mutation (Karmon et al. 2022).

Given that ADNP/NAP and cocaine are known to interact with MT-EB proteins, it is possible that the cocaine-ADNP connection is mediated through the microtubular system. Cocaine primarily acts on monoamine transporters, raising synaptic dopamine (DA), norepinephrine, and serotonin levels by blocking neurotransmitter reuptake (Solinas et al 2019). Among its many downstream effects, cocaine-induced increases in DA lead to activation of Arc (activity-regulated cytoskeletal-associated protein) (Tan et al. 2000), as well as overexpression of dopamine transporter (DAT) and alpha synuclein (Mash et al. 2008). Overexpression of alpha synuclein is associated with impaired microtubule-dependent trafficking and assembly, as well as Tau phosphorylation and aggregation (Oikawa et al 2016). Cocaine has been shown to increase levels of phosphorylated Tau in both cocaine-treated rats (Liu et al. 2003) and in the post-mortem brains of young drug abusers (Ramage et al. 2005).

ADNP is protective against dopamine and 6-OHDA toxicity *in vitro* (Offen et al. 2000), and NAP has been shown to reduce Tau phosphorylation* in vivo* (Shiryaev et al. 2009). Moreover, in mice overexpressing alpha synuclein, NAP treatment improved behavioral deficits, recovered motor function, and decreased both alpha synuclein inclusions and Tau hyperphosphorylation (Fleming et al. 2011; Magen and Gozes 2013). A complementary mechanism may involve actin, with cocaine inactivating cofilin, a primary regulator of the neuronal actin cytoskeleton (Sequeira et al. 2023), and with ADNP including an actin binding site (Ivashko-Pachima et al. 2022). Additional shared cocaine-ADNP pathways may involve Wnt/beta-catenin signaling (Cuesta et al. 2017a, b; Sun et al. 2020), SIRT1 activation (Ferguson et al. 2015; Hadar et al. 2021), and autophagy (Guo et al. 2015; Amram et al. 2016).

A pattern for all drugs of abuse is that females begin to use drugs at lower doses than males, but their use escalates more rapidly, and it is more difficult for females to quit once addicted (Fattore et al. 2020). Women show an enhanced response to cocaine, and abstinent women report higher levels of craving when exposed to cocaine-related cues (Becker 2016; Robbins et al. 1999). From a neurobiological perspective, these differences are driven by both gonadal hormones and sex chromosome complement (Knouse and Briand 2021; Hu et al. 2004) (for a full review of sex differences in cocaine use disorder, see Becker 2016; Fattore et al. 2008; Harp, et al. 2020; Kokane and Perrotti 2020 Peart et al. 2022).

The ovarian hormone estradiol (E2) is crucial in mediating behavioral and physiological responses to cocaine (Peart et al. 2022; Justice and Wit 2000), including sensitivity to the drug and symptoms of withdrawal/craving. Estradiol regulates structural plasticity through multiple pathways, including activation of metabotropic glutamate receptor type 5 (mGluR5), the extracellular signal-regulated kinase/mitogen activated protein kinase (ERK/MAPK) pathway, cyclic AMP response element binding protein (CREB), and activation of the mammalian target of rapamycin (mTOR) protein synthesis pathway (Lacy et al. 2016; Knouse and Briand 2021).

Significantly, ADNP is a vasoactive intestinal peptide (VIP)-responsive gene, and VIP is regulated by estrogen in the hypothalamus (Gozes et al. 1989; Gozes 2024). ADNP levels in hypothalamus and arcuate nucleus are mediated by the estrous cycle, with pro-estrous sections being the most ADNP-immunoreactive (Furman et al. 2005). As highlighted in depth earlier in this paper, ADNP is a sexually dimorphous gene. Our discovery that the ADNP gene mediates some effects of cocaine on spine morphology add to our understanding of structural mechanisms underlying sex differences in CUD. We suggest future studies using the *Adnp*^*+/−*^ animal model to test the relationship between sex- cocaine use- structural plasticity.

Clinically, the ADNP-cocaine connection is especially relevant for individuals with deregulated ADNP, i.e., patients with autism spectrum disorder and patients with schizophrenia. Both populations may be at risk for substance abuse disorders (Ressel et al. 2020; Winklbaur et al. 2006) and present additional challenges in treatment (De Witte et al. 2014; Helverschou et al. 2019).

More broadly, individuals with pre-existing cognitive deficits and/or comorbid conditions affecting cognitive function are more vulnerable to drug-abuse (Majewska 1996; D’Souza 2019). Cocaine can worsen pre-existing and induce new cognitive impairments, both of which are correlated with decreased neurogenesis (D’Souza 2019). In addition, repetitive administration and high, short-term doses impair hippocampal neurogenesis (Yamaguchi et al. 2004; Sudai et al. 2011), though if taken acutely, cocaine can improve hippocampal function (Thompson et al. 2002) and prospective memory (Hutten et al. 2018). Conversely, promoting hippocampal neurogenesis in rats has been shown to reduce the rates of drug use and relapse (Noonan et al. 2010; Deschaux et al. 2014). These and related findings suggest that enhancing neurogenic activity and/or cognitive function could be one approach for treating CUD (Mash et al. 2008). For example, modafinil is a cognitive enhancer that has been tested as a treatment option (Anderson et al. 2009; Brandt et al. 2021; Buchholz and Saxon 2019; Sangroula et al. 2017).

Another drug that shows promise for substance use disorders, and CUD in particular, is ketamine (Gao et al. 2023). Ketamine modulates glutamatergic signaling through N-methyl-D-aspartate (NMDA) receptor antagonism and is used medically as an aesthetic. At low doses, it has been suggested as a therapeutic treatment for psychiatric conditions including depression (Jawad et al. 2023; Tsang et al. 2023), chronic pain (Riccardi et al. 2023), post-traumatic stress disorder (Asim et al. 2021), as well as autism spectrum disorder (Wink et al. 2014). Conversely, exposure to ketamine in early postnatal periods can lead to ASD symptoms in adult mice (Hadar et al. 2021) and used recreationally, ketamine has a high potential for addiction. Other ketamine limitations have been recently discussed (Ganaiem et al. 2023).

In this study, we show that cocaine binds to a Zn finger adjacent to the NAP binding site. Interestingly, this is the same site that ketamine binds to as well (Ganaiem et al. 2023). Our findings that cocaine and NAP have similar effects on synaptic plasticity, together with our *in silico* results highlighting the overlap of cocaine/ketamine NAP binding, indicate that NAP could be a safe (Hacohen-Kleiman et al. 2018) non-addictive alternative to investigate as a potential treatment for CUD, in a sex-dependent manner.

Lastly, addressing future studies, the autistic ADNP syndrome shows Alzheimer’s disease-like tauopathy (Grigg et al. 2020), which is protected against by NAP treatment (Karmon et al. 2022). Mechanistically, NAP binding to EB1/EB3 enhances Tau-MT interactions (Ivashko-Pachima et al. 2017, 2021) and protects against Tau hyperphosphorylation and Tau tangle-like depositions (Karmon et al. 2022). Conversely, cocaine was suggested to induce Tau hyperphosphorylation leading to tauopathy (Liu et al. 2003), paralleled by ADNP changes in the face of tauopathy (Schirer et al. 2014). Taken together, the findings imply compensation/replacement by NAP in CUD.

## Conclusion

In conclusion, our findings suggest that ADNP is a potential novel biomarker and regulator of cocaine addiction, at the synaptic level. Future studies addressing the mechanisms downstream of ADNP will be beneficial to understanding the regulatory role of ADNP on cocaine-related behaviors and pathology. Measurements of ADNP levels, predictive of cognitive abilities (Malishkevich et al. 2016), coupled with NAP therapy in a dose- and sex-dependent manner may provide a potential treatment for CUD, especially when cognitive dysfunction is detected (Mahoney 2019).

## Data Availability

No datasets were generated or analyzed during the current study.
